# The microRNA expression signature of CD4+ T cells in the transition of brucellosis into chronicity

**DOI:** 10.1371/journal.pone.0198659

**Published:** 2018-06-13

**Authors:** Ferah Budak, Salih Haldun Bal, Gulcin Tezcan, Emin Halis Akalın, Abdullah Yılmaz, Pınar Hız, Haluk Barbaros Oral

**Affiliations:** 1 Department of Immunology, Faculty of Medicine, Uludag University, Bursa, Turkey; 2 Institute of Fundamental Medicine and Biology, Kazan Federal University, Kazan, Russia; 3 Department of Infectious Diseases and Clinical Microbiology, Faculty of Medicine, Uludag University, Bursa, Turkey; 4 Department of Immunology, Aziz Sancar Institute of Experimental Medicine, Istanbul University, Istanbul, Turkey; East Carolina University Brody School of Medicine, UNITED STATES

## Abstract

Brucellosis is a serious infectious disease that continues to be a significant cause of morbidity worldwide and across all ages. Despite early diagnosis and treatment, 10–30% of patients develop chronic brucellosis. Although there have been recent advances in our knowledge of Brucella virulence factors and hosts’ immune response to the infection, there is a lack of clear data regarding how the infection bypasses the immune system and becomes chronic. The present study investigated immunological factors and their roles in the transition of brucellosis from an acute to a chronic infection in CD4+ T cells. CD4+ T cells sorted from peripheral blood samples of patients with acute or chronic brucellosis and healthy controls using flow cytometry as well as more than 2000 miRNAs were screened using the GeneSpring GX (Agilent) 13.0 miRNA microarray software and were validated using reverse transcription polymerase chain reaction (RT-qPCR). Compared to acute cases, the expression levels of 28 miRNAs were significantly altered in chronic cases. Apart from one miRNA (miR-4649-3p), 27 miRNAs were not expressed in the acute cases (p <0.05, fold change> 2). According to KEGG pathway analysis, these miRNAs are involved in the regulation of target genes that were previously involved in the MAPK signalling pathway, regulation of the actin cytoskeleton, endocytosis, and protein processing in the endoplasmic reticulum. This indicates the potential role of these miRNAs in the development of chronic brucellosis. We suggest that these miRNAs can be used as markers to determine the transition of the disease into chronicity. This is the first study of miRNA expression that analyses human CD4+ T cells to clarify the mechanism of chronicity in brucellosis.

## Introduction

Brucellosis is one of the most common diseases among humans and animals. The disease is caused by bacteria of the *Brucella* genus. These bacteria are facultative, intracellular, Gram-negative, non-spore-forming, non-motile coccobacilli that infect humans as well as domestic mammals (cattle, sheep, goat, swine, etc.) and wild mammals (deer, bison, etc.) [1]. Humans can become infected in different ways, including through direct contact with animals infected with *Brucella* and consumption of products derived from animals infected with *Brucella*. The majority of human cases are caused by *Brucella melitensis*, although *Brucella abortus*, *Brucella suis*, *Brucella canis*, and, more recently, *Brucella pinnipedialis* and *Brucella ceti* have also been associated with the acquisition of brucellosis in humans [2, 3]. In humans, the *Brucella* infection is considered either a generalized, febrile illness that does not impact organ systems or a focal disease in which one or more organs are involve, such as in the most common form of the disease-osteoarticular brucellosis [4, 5].

In acute brucellosis patients, the acute phase of the disease follows the eradication of the bacteria by the immune system [6]. However, despite early diagnosis and treatment, a failure to clear the brucellosis infection in its acute form leads the disease to transition to its chronic form, which is observed in almost 10–30% of patients [7, 8]. The diagnosis of chronicity is mainly based on clinical symptoms and high IgG titres in serological tests [9]. However, IgG titres may remain positive for years following the successful resolution of symptoms because of low specificity of the assay [7, 10]. Therefore, markers are needed for the clinical prediction, accurate diagnosis, and follow-up of brucellosis and for administering successful therapeutic approaches. Additionally, the mechanisms that lead to chronicity in brucellosis are not completely established. To understand the mechanism by which the infection evades the host’s immune response, we previously evaluated the molecular markers in the peripheral blood mononuclear cells (PBMCs) and CD8 (+) T lymphocytes of patients with acute and chronic brucellosis [11, 12]. However, the molecular markers expressed in CD4 (+) T lymphocytes remain unknown.

CD4+ T cells play a major role in regulating the immune system by helping to activate the cells of the innate immune system and B-cells and helping to generate cytotoxic CD8+ T cells and nonimmune cells. Moreover, CD4+ T cells play critical role in the suppression of immune reactions [13]. Several studies have identified new subsets of CD4^+^ cells besides the well-known T-helper 1 (T_H_1) and T-helper 2 (T_H_2) cells. T_H_1 cells are involved in host defence against intracellular pathogens and tumour cells. T_H_2 cells are responsible for coordinating humoral immunity and eosinophilic inflammation and are involved in host defence against extracellular parasites. New subsets of CD4+ cells include T-helper (T_H_9), T-helper 17 (T_H_ 17), T-helper 22 (T_H_22), follicular helper T cell (T_H_FH), Natural Killer T cell (NKT), induced T-regulatory cells (iTreg), regulatory type 1 cells (Tr1), and CD4+ Cytotoxic T cells (CD4+ CTL) [14–16]. Studies have shown that IFN-γ-mediated T_H_1 responses are essential for the clearance of intracellular bacteria such as *Brucella* spp. [17]. In a recent study, Martirosyan et al. show that, with high levels of IFN-γ and granzyme B expression, CD4+ CTL participated in the early phase clearance of the *Brucella* infection [16]. Thus, to provide valuable information regarding the pathogenesis of brucellosis and to develop new therapeutic approaches for treating brucellosis and preventing the transition to the chronic form of the disease, the molecular markers of all immune system components related to brucellosis must be identified.

MicroRNAs (miRNAs) are small, non-coding RNAs of 18–25 nucleotides in length that bind to complementary UTR regions of target mRNAs and regulate the transcriptional activity of the target gene [18, 19]. miRNAs have been implicated in several basic metabolic pathways and some biological processes, such as the regulation of haematopoiesis, cellular proliferation, and programmed cell death. Differentiation, organogenesis, and abnormalities in these miRNAs contribute to the development of diseases, such as cancerogenesis, and infections [20–26].

In our previous studies, we determined the changes in the miRNA expression of the PBMCs and CD4+ T cells that play important roles in the establishment and clearance of chronic *Brucella* infections [11, 12]. In this study, we aimed to investigate the potential miRNA biomarkers in CD4+ T cells that indicate whether a case of the *Brucella* infection turns into chronic form. To this end, the expression changes of the miRNAs involved in the immune responses of CD4+ T cells were comparatively examined in patients with acute and chronic brucellosis as well as healthy controls.

## Materials and methods

### Ethics statement

This study protocol was approved by the Committee on the Ethics of the University of Uludag, Faculty of Medicine (Permit number:2010-6/2). All participiants gave written informed consent for inclusion.

### Patient cohort

A total of 15 patients with brucellosis (9 female and 6 male) with bone and joint involvement served as the experimental group, and a total of 8 healthy volunteers (4 female and 4 male) served as the control group. A diagnosis of brucellosis was made with the existence of one or more constitutional symptoms, such as fever, perspiration, and fatigue, as well as with the standard tube agglutination test titre in the Clinical Bacteriology and Infectious Diseases Department at Uludag University Faculty of Medicine [6]. Exclusion criteria were chronic granulomatous disease other than brucellosis, other infectious diseases, cardiovascular diseases, chronic renal insufficiency, chronic liver diseases, malignancy, collagen tissue disease, autoimmune disease. All cases were treated with the same therapy protocol as described previously [11]. Chronic brucellosis cases were followed up in outpatient clinic at least for 12 months. The duration of treatment for chronic brucellosis was three months and extended according to patient’s clinical status. The study was approved by the local ethics committee (approval number: 2010-6/2) and complied with the ethical standards of the Helsinki Declaration. All participants received information about the study, and all patients provided informed consent to participate in the study.

### CD4+ T cell isolation

The CD4+ T cells of the cases were obtained from PBMCs, which were separated from 20 mL of peripheral blood using an intensity gradient centrifuge method with Ficoll (Biochrom, Germany). CD4+ T cells were sorted from the suspension of PBMCs using FACSAria (Beckton Dickinson [BD] Biosciences Cell-sorter, USA) after the cells were stained with CD4-FITC, CD8-PE, and CD3-PerCP antibodies (BD, Biosciences, USA). Briefly, live PBMCs were gated based on forward versus side-scatter profiles, followed by CD3+ based gating for T lymphocytes were identified. The CD3+ positive population was subdivided by CD8/CD4 expression and used to identify CD4+ (CD4+, CD8-) T cells. The purity of the resulting CD4+ T cells was 99.8%.

### Microarray analysis

miRNA was isolated from CD4+ T cells using an miRNeasy Kit (Qiagen, Germany) and was labelled with Cy3 during transformation into cDNA using an RNA Agilent miRNA Labeling Kit (Agilent, UK) and Spike Kit (Agilent, UK). cDNAs were hybridized to Human miRNA Microarray, Release 19.0, 8x60K(v19) microarray slides (Agilent, UK) according to Agilent microRNA Hybridization Kit protocol (Agilent, UK) in duplicate and scanned using an Nimblegen MS200 array scanner. Data were normalized using the Plier algorithm and were log transformed and analysed using GeneSpring GX 13.0 (Agilent, UK). Significant miRNAs were determined based on P-values < 0.05 or fold changes in the range of >2 and <-2.

Data availability: The microarraydata that supported the findings of this study were deposited in the Gene Expression Omnibus with an accession number of GSE107554.

### qRT-PCR

Microarray analyses were validated using qRT-PCR for ten randomly selected miRNAs using the same miRNA stock samples isolated for microarray analysis. Using a Universal cDNA Synthesis Kit (Exiqon, Denmark), 5ng/μL of miRNA samples were reverse transcribed into cDNA. qRT-PCR was performed using LightCycler 480 II (Roche, Germany) microRNA LNA^™^ primer sets and an ExiLENT SYBR1 Green Master Mix Kit (Exiqon, Denmark), as described previously [11].

### Identification of miRNAs, target proteins, and related pathways

Biological potency and functional classification of predicted miRNA targets were identified using pathway enrichment analysis. Selected miRNAs were entered into KEGG (Kyoto Encyclopedia of Genes and Genomes) (http://www.genome.jp/kegg/pathway.html) to elucidate the number of predicted miRNA target genes [27]. The signalling pathways associated with the predicted target genes of the miRNAs were determined using a web-based enrichment analysis tool known as WebGestalt (WEB-based GEneSeTAnaLysis Toolkit) (http://bioinfo.vanderbilt.edu/webgestalt/) and KEGG [27, 28].

## Results

### Clinical data

Based on the onset time of the symptoms, 7 of the cases (3 female and 4 male) whose onset was in a range of 0–2 months were diagnosed with acute brucellosis, and 8 of the cases (6 female and 2 male) whose onset was >12 months were diagnosed with chronic brucellosis [29, 30]. The median age of cases with acute brucellosis was 52.14 ±10.40, and the median age of cases with chronic brucellosis was 45.00 ±11.48. The median age of the control group was 39.62 ± 7.74.

### miRNA expression pattern of CD4+ T cells in brucellosis

**Fig 1:**
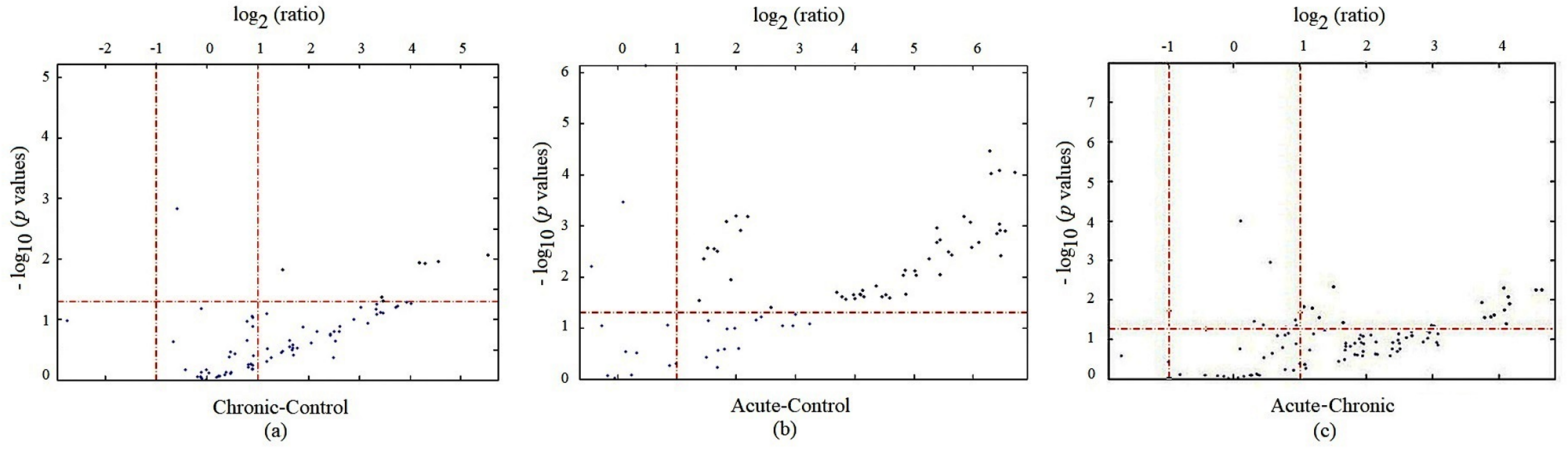
Differential expressions of miRNAs evaluated in (a) chronic versus control, (b) acute versus control, and (c) acute versus chronic cases depend on miRNA microarray analysis (p<0.05, cut off = 2).

According to the microarray analyses, miRNAs with more than twofold differences between the samples and control were evaluated, and those that expressed with less than twofold differentiation were assumed as negligible (Fig 1). Based on the results of an independent samples t-test, 11 miRNAs were altered in all brucellosis cases (whether acute or chronic) compared to the control cases (Table 1). Besides these 11 miRNAs, the expression levels of the 41 miRNAs were different from those of the control cases, but these miRNAs demonstrated distinct expression patterns between the acute and chronic brucellosis cases (Table 2).

**Table 1:** List of significantly altered miRNA expressions in both acute and chronic brucellosis cases compared to control cases.

	Chronic vs Control			Acute vs Control			Chronic vs Acute		
miRNA	Fold change	Fold regulation	P value*	Fold change	Fold regulation	p value*	Fold change	Fold regulation	P value*
hsa-miR-4530	46.11	46.11	0.0085	88.44	88.44	0.0037	0.52	-1.92	0.1294
hsa-miR-4739	18.12	18.12	0.0113	17.18	17.18	0.0225	1.05	1.05	0.9648
hsa-miR-762	14.67	14.67	0.0114	27.37	27.37	0.0091	0.54	-1.87	0.6060
hsa-miR-4787-5p	8.15	8.15	0.0629	15.74	15.74	0.0220	0.52	-1.93	0.0459
hsa-miR-940	8.02	8.02	0.0337	10.45	10.45	0.0309	0.77	-1.30	0.8215
hsa-miR-3676-5p	4.19	4.19	0.1823	3.11	3.11	0.1725	1.35	1.35	0.8099
hsa-miR-6090	3.70	3.70	0.1313	5.05	5.05	0.0695	0.73	-1.37	0.2925
hsa-miR-150-5p	3.09	3.09	0.2224	3.24	3.24	0.2746	0.95	-1.05	0.9478
hsa-miR-4516	2.82	2.82	0.0151	4.22	4.22	0.0012	0.67	-1.50	0.2302
hsa-miR-4284	2.41	2.41	0.4221	4.13	4.13	0.2489	0.58	-1.72	0.5832
hsa-miR-3656	2.29	2.29	0.3029	3.94	3.94	0.0998	0.58	-1.72	0.0486

*P values were calculated using an independent samples t-test.

**Table 2:** List of miRNAs that were differently regulated between acute and chronic brucellosis.

	Chronic vs Control			Acute vs Control			Chronic vs Acute		
miRNA	Fold change	Fold regulation	P value*	Fold change	Fold regulation	P value*	Fold change	Fold regulation	P value*
hsa-miR-6068	23.45	23.45	0.0110	86.89	86.89	0.0009	0.27	-3.70	0.0975
hsa-miR-4497	19.62	19.62	0.0117	86.96	86.96	8.15E-05	0.23	-4.43	0.1161
hsa-miR-1207-5p	16.26	16.26	0.0538	63.06	63.06	0.0026	0.26	-3.88	0.2013
hsa-miR-197-5p	15.29	15.29	0.0517	92.97	92.97	0.0012	0.16	-6.08	0.0920
hsa-miR-642a-3p	13.53	13.53	0.0591	87.52	87.52	0.0012	0.15	-6.47	0.0817
hsa-miR-3162-5p	13.16	13.16	0.0627	84.64	84.64	0.0014	0.16	-6.43	0.0818
hsa-miR-4687-3p	11.03	11.03	0.0489	61.77	61.77	0.0008	0.18	-5.60	0.0724
hsa-miR-3196	10.87	10.87	0.0421	41.87	41.87	0.0020	0.26	-3.85	0.2665
hsa-miR-371b-5p	10.65	10.65	0.0768	68.32	68.32	0.0020	0.16	-6.42	0.0650
hsa-miR-3665	10.15	10.15	0.0807	32.77	32.77	0.0092	0.31	-3.23	0.1531
hsa-miR-4466	10.12	10.12	0.0551	38.25	38.25	0.0044	0.26	-3.78	0.0781
hsa-miR-574-5p	10.05	10.05	0.0673	32.45	32.45	0.0075	0.31	-3.23	0.1254
hsa-miR-1225-5p	8.96	8.96	0.1155	49.89	49.89	0.0037	0.18	-5.57	0.1063
hsa-miR-4485	8.60	8.60	0.0341	25.67	25.67	0.0087	0.33	-2.99	0.3709
hsa-miR-572	7.78	7.78	0.0332	40.92	40.92	0.0010	0.19	-5.26	0.1215
hsa-miR-5001-5p	7.46	7.46	0.0984	28.89	28.89	0.0073	0.26	-3.87	0.0857
hsa-miR-4672	6.15	6.15	0.1297	104.31	104.31	8.96E-05	0.06	-16.95	0.0182
hsa-miR-1234-5p	6.09	6.09	0.1573	20.53	20.53	0.0149	0.30	-3.37	0.1528
hsa-miR-4465	6.01	6.01	0.0798	50.85	50.85	0.0086	0.12	-8.46	0.1389
hsa-miR-4299	5.78	5.78	0.2277	43.53	43.53	0.0091	0.13	-7.53	0.0606
hsa-miR-6132	5.67	5.67	0.1578	29.19	29.19	0.0218	0.19	-5.15	0.2663
hsa-miR-4763-3p	5.37	5.37	0.1745	43.50	43.50	0.0018	0.12	-8.10	0.0465
hsa-miR-937-5p	5.36	5.36	0.1815	17.03	17.03	0.0219	0.31	-3.17	0.3272
hsa-miR-5585-3p	4.98	4.98	0.0809	26.03	26.03	0.0087	0.19	-5.23	0.1785
hsa-miR-3679-5p	4.48	4.48	0.1570	78.89	78.89	9.51E-05	0.06	-17.61	0.0083
hsa-miR-3940-5p	4.26	4.26	0.0797	71.52	71.52	5.56E-08	0.06	-16.79	0.0050
hsa-miR-6088	4.16	4.16	0.2430	17.49	17.49	0.0181	0.24	-4.20	0.0799
hsa-miR-638	3.43	3.43	0.2978	13.00	13.00	0.0200	0.26	-3.79	0.1218
hsa-miR-6127	3.24	3.24	0.3862	47.74	47.74	0.0032	0.07	-14.72	0.0268
hsa-miR-4486	3.22	3.22	0.2627	6.86	6.86	0.0910	0.47	-2.13	0.5447
hsa-miR-1202	3.22	3.22	0.2973	17.77	17.77	0.0245	0.18	-5.52	0.2000
hsa-miR-4428	3.21	3.21	0.3204	57.54	57.54	0.0006	0.06	-17.90	0.0124
hsa-miR-5787	3.11	3.11	0.1781	13.96	13.96	0.0328	0.22	-4.49	0.2420
hsa-miR-4721	3.10	3.10	0.2778	77.63	77.63	3.39E-05	0.04	-25.03	0.0055
hsa-miR-1973	2.85	2.85	0.1706	24.12	24.12	0.0092	0.12	-8.47	0.0725
hsa-miR-5739	2.82	2.82	0.3329	15.99	15.99	0.0264	0.18	-5.67	0.1735
hsa-miR-4532	2.82	2.82	0.1706	10.25	10.25	0.0304	0.28	-3.64	0.2504
hsa-miR-3651	2.77	2.77	0.3462	23.08	23.08	0.0221	0.12	-8.34	0.1226
hsa-miR-1275	2.65	2.65	0.1709	35.41	35.41	0.0011	0.07	-13.35	0.0117
hsa-miR-4728-5p	2.64	2.64	0.1705	20.48	20.48	0.0092	0.13	-7.76	0.0682
hsa-miR-1915-3p	2.26	2.26	0.4917	7.97	7.97	0.0543	0.28	-3.53	0.1282

*P values were calculated using an independent samples t-test.

### Unique miRNA expression pattern of CD4+ T cells in acute or chronic forms of brucellosis

Twenty-eight miRNAs were significantly altered in the chronic cases. Twenty-seven of these miRNAs were not expressed in the acute cases, and one of them (miR-4649-3p) did not demonstrate significant differences between the acute cases and control cases (Table 3). However, 18 miRNAs were significantly altered in the acute cases. The expressions of these miRNAs were similar in the chronic cases and control cases (Table 4).

**Table 3:** List of significantly altered miRNA expressions in chronic brucellosis cases.

	Chronic vs Control			Acute vs Control			Chronic vs Acute		
miRNA	Fold change	Fold regulation	P value*	Fold change	Fold regulation	P value*	Fold change	Fold regulation	P value*
hsa-miR-4649-3p	5.51	5.51	0.0859	1.70	1.70	0.3559	3.24	3.24	0.2660
hsa-miR-885-5p	2.16	2.16	0.3506	-	-	-	2.16	2.16	0.3506
hsa-miR-766-3p	2.02	2.02	0.3506	-	-	-	2.02	2.02	0.3506
hsa-miR-6511b-3p	2.04	2.04	0.3506	-	-	-	2.04	2.04	0.3506
hsa-miR-6511a-3p	2.13	2.13	0.3506	-	-	-	2.13	2.13	0.3506
hsa-miR-5584-3p	2.02	2.02	0.3506	-	-	-	2.02	2.02	0.3506
hsa-miR-483-3p	2.09	2.09	0.3506	-	-	-	2.09	2.09	0.3506
hsa-miR-4763-5p	2.06	2.06	0.3506	-	-	-	2.06	2.06	0.3506
hsa-miR-4695-3p	2.08	2.08	0.3506	-	-	-	2.08	2.08	0.3506
hsa-miR-449b-3p	2.07	2.07	0.3506	-	-	-	2.07	2.07	0.3506
hsa-miR-4436b-5p	2.03	2.03	0.3506	-	-	-	2.03	2.03	0.3506
hsa-miR-3622b-3p	2.01	2.01	0.3506	-	-	-	2.01	2.01	0.3506
hsa-miR-3617-3p	2.12	2.12	0.3506	-	-	-	2.12	2.12	0.3506
hsa-miR-328	2.21	2.21	0.3506	-	-	-	2.21	2.21	0.3506
hsa-miR-3195	2.99	2.99	0.1716	-	-	-	2.99	2.99	0.1716
hsa-miR-2277-3p	2.02	2.02	0.3506	-	-	-	2.02	2.02	0.3506
hsa-miR-211-5p	2.09	2.09	0.3506	-	-	-	2.09	2.09	0.3506
hsa-miR-204-5p	2.05	2.05	0.3506	-	-	-	2.05	2.05	0.3506
hsa-miR-1976	2.02	2.02	0.3506	-	-	-	2.02	2.02	0.3506
hsa-miR-197-3p	2.11	2.11	0.3506	-	-	-	2.11	2.11	0.3506
hsa-miR-1825	2.86	2.86	0.1727	-	-	-	2.86	2.86	0.1727
hsa-miR-1296	2.01	2.01	0.3506	-	-	-	2.01	2.01	0.3506
hsa-miR-129-1-3p	3.07	3.07	0.1705	-	-	-	3.07	3.07	0.1705
hsa-miR-1281	2.87	2.87	0.1729	-	-	-	2.87	2.87	0.1729
hsa-miR-1260a	2.09	2.09	0.3506	-	-	-	2.09	2.09	0.3506
hsa-let-7e-3p	2.06	2.06	0.3506	-	-	-	2.06	2.06	0.3506
hsa-let-7d-3p	2.11	2.11	0.3506	-	-	-	2.11	2.11	0.3506
hsa-let-7b-3p	3.22	3.22	0.1791	-	-	-	3.22	3.22	0.1791

*P values were calculated using independent sample T test.

**Table 4:** List of significantly altered miRNA expressions in acute brucellosis cases.

	Chronic vs Control			Acute vs Control			Chronic vs Acute		
miRNA	Fold change	Fold regulation	P value*	Fold change	Fold regulation	P value*	Fold change	Fold regulation	P value*
hsa-miR-939-5p	-	-	-	5.64	5.64	0.0782	0.18	-5.64	0.0782
hsa-miR-6724-5p	1.85	1.85	0.6528	28.13	28.13	0.0092	0.07	-15.21	0.0244
hsa-miR-6125	1.38	1.38	0.3405	2.87	2.87	0.0027	0.48	-2.08	0.0152
hsa-miR-6075	-	-	-	9.40	9.40	0.0303	0.11	-9.40	0.0303
hsa-miR-5006-5p	-	-	-	2.03	2.03	0.3559	0.49	-2.03	0.3559
hsa-miR-494	1.87	1.87	0.1288	4.57	4.57	0.0006	0.41	-2.44	0.0283
hsa-miR-4746-3p	1.87	1.87	0.3506	10.54	10.54	0.0311	0.18	-5.65	0.1253
hsa-miR-4665-3p	-	-	-	2.89	2.89	0.1723	0.35	-2.89	0.1723
hsa-miR-4507	1.38	1.38	0.7716	5.37	5.37	0.0608	0.26	-3.89	0.1322
hsa-miR-4459	1.74	1.74	0.2177	3.99	3.99	0.0006	0.44	-2.30	0.0739
hsa-miR-4430	1.74	1.74	0.3506	23.90	23.90	0.0106	0.07	-13.77	0.0281
hsa-miR-4281	1.87	1.87	0.5650	6.00	6.00	0.0400	0.31	-3.21	0.1870
hsa-miR-3653	1.40	1.40	0.7411	24.11	24.11	0.0257	0.06	-17.28	0.0400
hsa-miR-342-3p	0.98	-1.02	0.9800	3.48	3.48	0.2577	0.28	-3.55	0.2463
hsa-miR-3135b	1.81	1.81	0.5380	13.81	13.81	0.0247	0.13	-7.61	0.0958
hsa-miR-2861	1.36	1.36	0.4150	3.10	3.10	0.0028	0.44	-2.28	0.0161
hsa-miR-1587	1.89	1.89	0.3963	3.60	3.60	0.1031	0.53	-1.90	0.0331
hsa-let-7b-5p	1.76	1.76	0.5515	7.74	7.74	0.0893	0.23	-4.41	0.2377

*P values were calculated using an independent samples t-test.

### Predicted target pathways of miRNA

**Fig 2:**
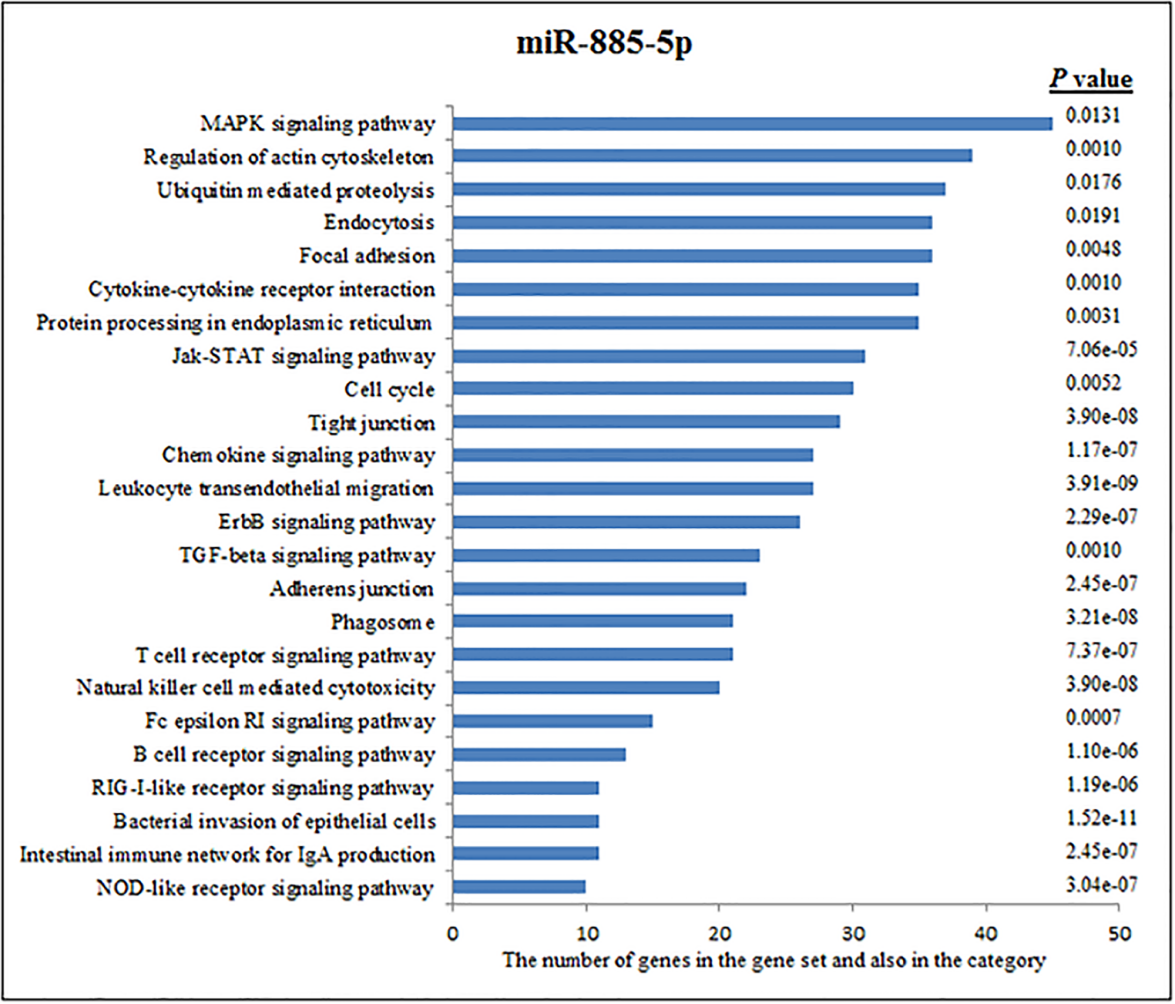
Pathway analysis of miR-885-5p according to KEGG function annotations.

**Fig 3:**
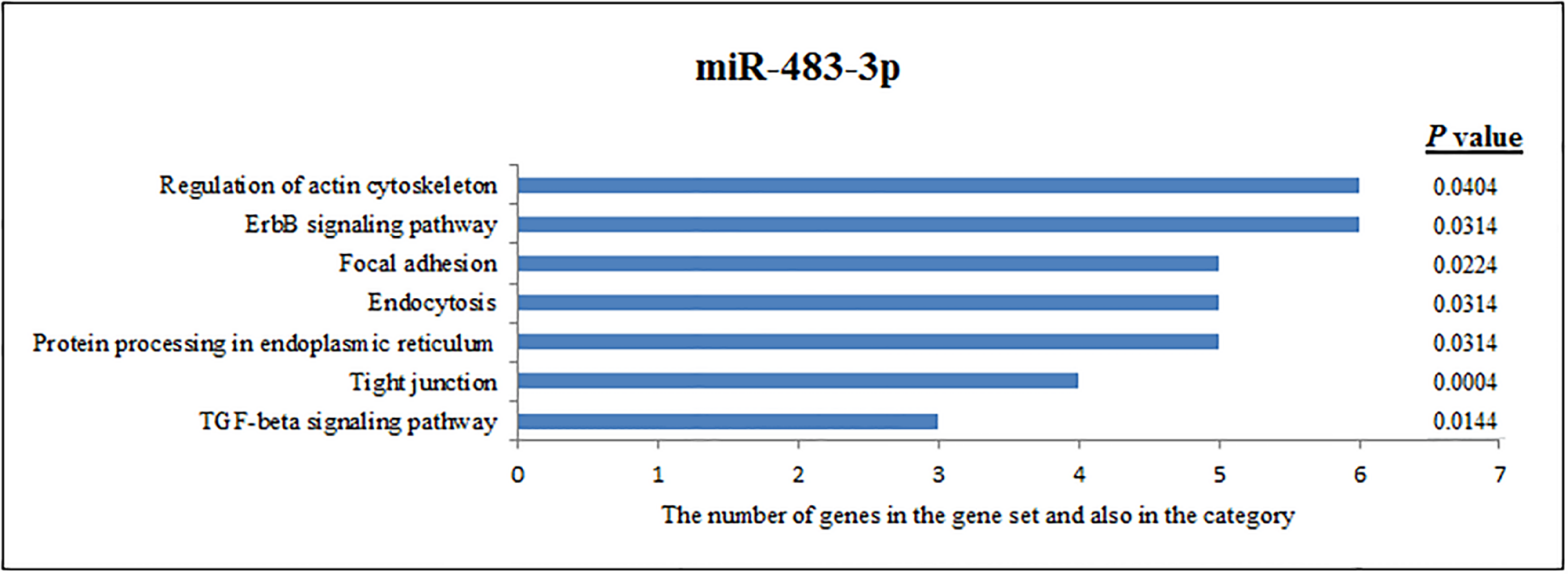
Pathway analysis of miR-483-3p according to KEGG function annotations.

**Fig 4:**
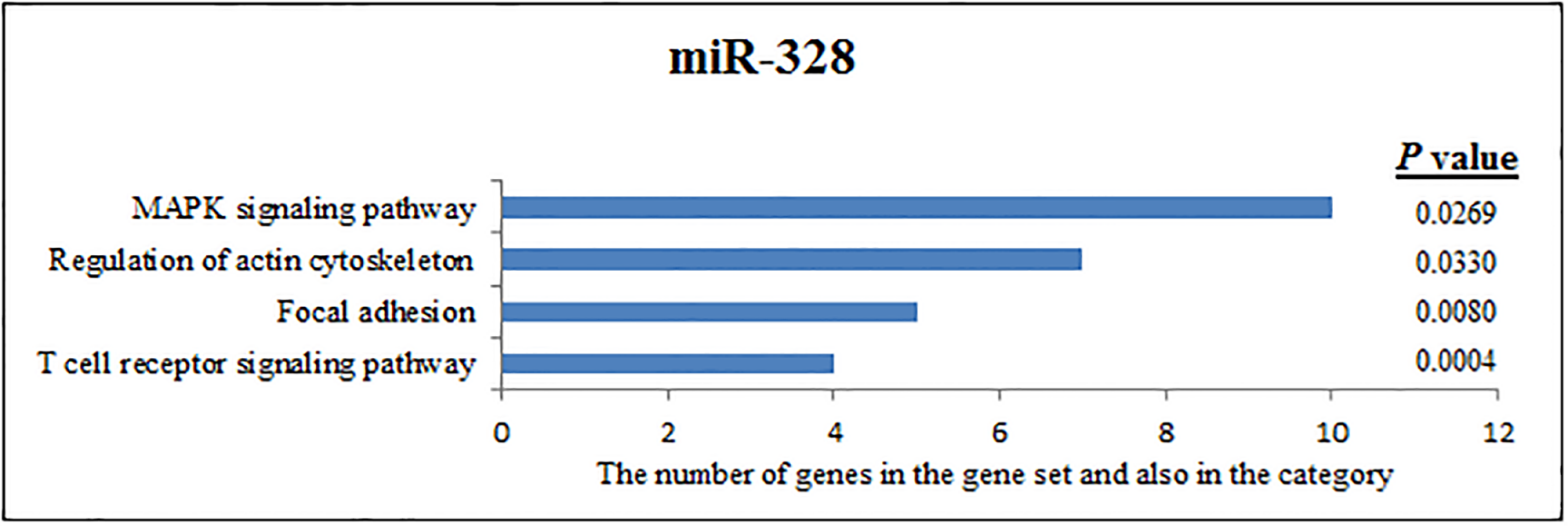
Pathway analysis of miR-328 according to KEGG function annotations.

The predicted target genes of miRNAs that uniquely expressed in the chronic cases, including miR-885-5p, miR-483-3p, and miR-328, were analysed. The target genes of these miRNAs and the immunologically effective pathways that are potentially manipulated by these miRNAs are presented in Figs 2–4. According to these analyses, miR-885-5p is involved in the regulation of 3085 gene expressions. According to KEGG function annotations, these mutual genes play a role in several signalling pathways, such as the MAPK signalling pathway, regulation of the actin cytoskeleton, ubiquitin-mediated proteolysis, endocytosis, focal adhesion, cytokine-cytokine receptor interactions, protein processing in the endoplasmic reticulum, the JAK-STAT signalling pathway, cell the cycle, tight junction, the chemokine signalling pathway, and leukocyte transendothelial migration pathways (Fig 2).

The results showed that 165 predicted genes were annotated by miR-483-3p. These genes have functions in the regulation of the actin cytoskeleton, the ErbB signalling pathway, focal adhesion, endocytosis, protein processing in the endoplasmic reticulum, tight junction, and the TGF-beta signalling pathway (Fig 3).

According to KEGG function annotations, 332 predicted genes have a binding site for miR-328. These genes are involved in the MAPK signalling pathway, regulation of the actin cytoskeleton, focal adhesion, and T cell receptor signalling pathway (Fig 4).

Moreover, not only were 41 miRNA expressions different from the control group, but they also displayed a difference between the acute and chronic brucellosis groups (Table 2).

Compared to the acute cases, the expression levels of 28 miRNAs were significantly altered in the chronic cases. All of these miRNAs were up-regulated in the chronic cases compared to in the control cases. Apart from 1 miRNA (miR-4649-3p), 27 miRNAs were not expressed in the acute cases (Table 3). In addition, the expression levels of 18 miRNAs were significantly altered in the acute cases. The expressions of these miRNAs were similar in the chronic cases and control cases (Table 4).

### Predicted target pathways of miRNA

To understand the role of immunological and genetic factors involved in the transition of brucellosis from an acute to a chronic infection, target pathway prediction of some miRNAs was performed according to KEGG function annotations. One increased miRNA (miR-885-5p) that targets genes of immunologically effective pathways is shown in Fig 2. Other increased miRNAs (miR-483-3p and miR-328) that target genes of immunologically effective pathways are shown in Figs 3 and 4, respectively.

Approximately 3,085 genes were regulated by miR-885-5p. According to KEGG function annotations, these mutual genes play a role in multiple signalling pathways, such as the MAPK signalling pathway, regulation of actin cytoskeleton, ubiquitin-mediated proteolysis, endocytosis, focal adhesion, cytokine-cytokine receptor interactions, protein processing in the endoplasmic reticulum, the JAK-STAT signalling pathway, the cell cycle, tight junction, the chemokine signalling pathway, and leukocyte transendothelial migration pathways (Fig 2).

The results showed that about 312 predicted genes were annotated by miR-483-3p. These genes are active in the regulation of the actin cytoskeleton, the ErbB signalling pathway, focal adhesion, endocytosis, protein processing in the endoplasmic reticulum, tight junction, and the TGF-beta signalling pathway (Fig 3).

The miR-328 that increased in the chronic cases was not expressed in the acute cases. The results showed that about 332 predicted genes were annotated with the genes MAPK signalling pathway, regulation of the actin cytoskeleton, focal adhesion and the T cell receptor signalling pathway (Fig 4).

## Discussion and conclusion

Brucellosis is a disease that is prevalent throughout the world. Although we know that brucellosis can convert from an acute to a chronic form, our understanding of the mechanisms of that transition remains incomplete. Multiple bacterial and host factors may be involved in this complex process [31]. Some studies have proposed that CD4+ T cells can play an important role in the regulation of the *Brucella* infection [32, 33]. Moreover, some studies have provided evidence that the gamma interferon (IFN-γ)-mediated T helper 1 (Th1) immune response is crucial for the regulation of the *Brucella* infection [34].

In the current study, we focused on miRNAs that are involved in the pathogenesis of the *Brucella* infection. The expression levels of these miRNAs demonstrate distinct patterns within individual cases across age, gender or clinical phenotype independent manner.

Analysing miRNA expressions in CD4+ T cells sorted from chronic, acute, and control cases, we investigated several miRNAs that are involved in the post-transcriptional regulation of gene expressions, which is associated with the immunological basis of chronicity.

So far, only a few studies have investigated the role of miRNAs in *Brucella* infections. One of these previous studies demonstrated the role of eight miRNAs in the *Brucella*–host interactions in-vitro conditions using mock- and *Brucella*-infected RAW264.7 cells and high-throughput sequencing. It was determined that the target genes of these miRNAs were involved in regulating apoptosis and autophagy [35].

Liu and his colleagues defined the role of miR-125b-5p-mediated A20 (TNFAIP3) regulation in macrophages activation of *B*. *abortus*-infection. According to their findings, reduced miR-125b-5p expression leads to increase in the A20 protein level, suppress the activation of NF-kB and leads to intracellular bacterial survival [36]. In another study, to identify miRNAs affected by the *Brucella* spp. infection in the absence CD14, Rong et al. analysed the miRNA expression profiles of CD14 knockdown RAW264.7 cells affected by *B*. *melitensis* M5-90 using an miRNA array. The authors observed up-regulation of mmu-miR-199a-3p and mmu-miR-183-5p. Interestingly, among the predicted target genes of mmu-miR-199a-3p and mmu-miR-183-5p, the significantly enriched gene ontology terms were the apoptosis process, immune response, inflammatory response, innate immune response, anti-apoptosis, cytokine production, and cytokine-mediated signalling pathway [37]. In one of our previous studies, more than 2,000 miRNAs were screened in peripheral blood mononuclear cells of patients with acute or chronic brucellosis, and we determined that while the expression level of miR-1238-3p increased, miR-494, miR-6069, and miR-139-3p decreased in the chronic group compared to the acute group. The involvement of differentially expressed miRNAs and their predicated target genes involved in endocytosis, regulation of actin cytoskeleton, the MAPK signaling pathway, and the cytokine-cytokine receptor interaction as well as its chemokine signaling pathway indicates the miRNAs’ potential roles in chronic brucellosis and its progression [11]. In another one of our previous studies, the regulatory roles of 2000 miRNA were evaluated in human CD8+ T cells. We determined that there are 42 miRNAs involved in the *Brucella* infection. Two of these miRNAs uniquely expressed in chronic brucellosis and five miRNAs uniquely expressed in acute brucellosis. The differentially expressed miRNAs in chronic brucellosis and their predicted target genes are involved in the MAPK signalling pathway, cytokine-cytokine receptor interactions, endocytosis, regulation of actin cytoskeleton, and focal adhesion [12].

The patients enrolled in the present study were treated with same antibiotic regimen of doxycycline and rifampicin. In two previous studies, the potential effect of rifampicin on the expression levels of miRNA was demonstrated [38, 39]. However, besides those two studies, the effects of antibiotics on miRNA expressions in other types of cells have not been examined. Because all the patients received the same antibiotic regimen, this issue was avoided in this study.

In the current study, the regulatory role of 2,000 miRNAs was evaluated in human CD4+ T cells. We determined that 11 miRNAs are involved in the *Brucella* infection. We also determined that 41 miRNAs were differently regulated between the acute and chronic brucellosis cases, 28 miRNAs were uniquely expressed in chronic brucellosis cases, and 18 miRNAs were uniquely expressed in acute brucellosis cases.

### Similarly expressed miRNAs in acute and chronic brucelosis

Eleven miRNAs (miRNA-4530, miRNA-4739, miRNA-762, miRNA-4787-5p, miRNA-940, miRNA-3676-5p, miRNA-6090, miRNA-150-5p, miRNA-4516, miRNA-4284, miRNA-3656) demonstrated a similar expression trend in both the acute and chronic groups. This indicated that both acute and chronic brucellosis may share, at least partly, similar regulatory mechanisms.

### Differently expressed miRNAs in acute brucellosis compared to in chronic brucellosis

In order to identify the miRNAs in PBMCs that may be correlated with chronicity, we focused on the differentially expressed miRNAs between the acute group and the chronic group. According to our findings, there were 41 dysregulated miRNAs between the two groups. According to our findings, all of the miRNAs were decreased in the chronic group compared with in the acute group.

Nineteen of the downregulated miRNAs in chronic patients were found to be associated with carcinogenesis in various organs and tissues, such as miR-1207 [40], miR-3162-5p [41, 42], miR-3196 [43, 44], miR-371b-5p [45], miR-574-5p [46, 47], miR-1225-5p [48], miR-4485 [49], miR-572 [50], miR-4299 [51], miR-3679-5p [52], miR-3940-5p [53], miR-638 [54, 55], miR-1202 [56], miR-5787 [57], miR-1973 [58], miR-4532 [59], miR-1275 [60], miR-4728-5p [61], and miR-1915-3p [62].

### Specifically altered miRNAs in chronic brucellosis

Determining the prognostic factors associated with the chronicity of brucellosis is necessary for developing follow-up treatments and improving alternative therapeutic protocols for treating the disease. In the current study, 28 miRNAs which displayed a statistically significant change in chronic cases compared to both the acute cases and the control cases were identified. All of the miRNAs were up-regulated in chronic cases compared to the control cases. Only miR-4649-3p was expressed in the acute cases, while the remaining 27 miRNAs were not.

One of the 28 miRNAs in which the expression was upregulated in chronic brucellosis is miR-885-5p. Afanasyeva et al. show that miR-885-5p inhibits cell proliferation and survival and promotes cellular senescence and apoptosis. They demonstrated that miR-885-5p downregulates cyclin-dependent kinase (CDK2) and that the mini-chromosome maintenance protein (MCM5) activates p53. They provided evidence that CDK2, which participates in cell cycle regulation and is especially critical during the G1 to S phase transition, and MCM5, which is involved in the initiation of DNA replication, are direct miR-885-5p targets [63]. In our study, this miRNA, which was only upregulated in the chronic group, may have played a significant role in the immunological response to *Brucella*. Yan et al. showed that miR-885-5p inhibited the expression of MMP9 indirectly [64]. MMM9 may play an essential role in the local proteolysis of the extracellular matrix, leukocyte migration, and bone osteoclastic resorption.

According to KEGG pathways analysis, miR-885-5p was linked to the MAPK signalling pathway, regulation of the actin cytoskeleton, ubiquitin-mediated proteolysis, endocytosis, focal adhesion, cytokine-cytokine receptor interactions, protein processing in the endoplasmic reticulum, the JAK-STAT signalling pathway, the cell cycle, tight junction, the chemokine signalling pathway, and leukocyte transendothelial migration pathways in chronic brucellosis.

Among the 28 miRNAs, the miR-483-3p upregulated in the chronic group in comparison to the control group. There was no expression in the acute infection group. Ni et al. showed that miR-483-3p is identified as a critical regulator of the expression of Insulin-like growth factor 1 (IGF-1) in natural killer cells [65]. According to present knowledge, IGF-1 is involved in the regulation of immunity and inflammation in several ways, such as regulating haematopoiesis, direct effector functions of cells of the innate and acquired immune systems, and functions of lymphocytes and monocytes via interactions with IGF-1R [65–68]. The majority of immune system cells carry the IGF-1R protein; thus, T lymphocytes are one of the targets of IGF-1 [66].

The function of IGF-1 in human natural killer cell development and cytotoxicity was demonstrated previously [65]. High miR-483-3p levels blockade IGF-1 and reduce natural killer cell cytotoxicity. These findings indicate that miR-483-3p is a regulator of natural killer cell functions [65]. According to our results that miR-483-3p upregulated in the chronic group compared to in the control group and that there was no expression in the acute group is remarkable. Dornand et al. showed that NK cells are activated by *B*. *suis*-infected macrophages and that they inhibit the intracellular multiplication of the bacteria by lysing the infected cells. Therefore, NK cells could be one actor for controlling the development of *Brucella* in humans [69].

One of the 28 miRNAs, miR-328, is involved in cancer [70, 71], autoimmune [72], and neuronal diseases [73]. Tay et al. demonstrated another role of miR-328 in the regulation of innate immune cell function by showing that suppression of miR-328 improved bacterial clearance in the lungs. According to their findings, miR-328 is involved in the regulation of phagocytosis and survival of bacteria through modifying the uptake of live, heat-killed, non-typeable *Haemophilus influenzae* [74]. They also suggested that bacterial killing enhanced by an oxygen-independent pathway depends on their increased cathepsin D observation in macrophages treated with anti-328 [74]. Thus, based on previous findings, miR-328 may have a strong potential to play a critical role in the microbial host defence of innate immune cells by augmenting phagocytosis as well as the production of ROS and microbicidal activity. In our study, the expression of miR-328 was 2.21-fold in the chronic group compared to in the control group. There was no expression in the acute group. Increased miR-328 levels in the chronic cases may be associated with fail of *Brucella* clearance and establishment of chronicity of *Brucella* infections. According to KEGG pathways analysis, miR-328 was linked to the MAPK signalling pathway, regulation of the actin cytoskeleton, focal adhesion, and the T cell receptor signalling pathway.

Identifying the prognostic factors of brucellosis is essential for ensuring more accurate prognoses as well as developing satisfactory follow-up and alternative therapeutic approaches to the disease. In the present study, we uniquely determined enhanced expressions of 28 miRNAs in CD4 (+) T cells which were significantly associated with chronicity in brucellosis. Due to these miRNAs identified in a small subset of individuals, the obtained data required to be validated in a larger group. In additions, further studies are needed to clarify the potential target genes and signalling pathways of these miRNAs and their relation to chronicity, but these preliminary findings suggest that the altered expression of these miRNAs in CD4 (+) T cells may serve as biomarkers that indicate progression to chronicity.
